# A k-mer based transcriptomics approach for antisense drug discovery targeting the Ewing’s family of tumors

**DOI:** 10.18632/oncotarget.25736

**Published:** 2018-07-17

**Authors:** Andrew J. Annalora, Shawn O’Neil, Jeremy D. Bushman, James E. Summerton, Craig B. Marcus, Patrick L. Iversen

**Affiliations:** ^1^ Department of Environmental and Molecular Toxicology, Oregon State University, Corvallis, OR 97331, USA; ^2^ Center for Genome Research and Biocomputing, Oregon State University, Corvallis, OR 97331, USA; ^3^ Onco-Tools, LLC, Philomath, OR 97370, USA; ^4^ LS Pharma, LLC, Grand Junction, CO 81507, USA

**Keywords:** antisense oligonucleotides, phosphorodiamidate morpholino oligomers, transcriptomics, Ewing’s family of tumors, RNA-based therapeutics

## Abstract

Ewing’s sarcoma treatment failures are associated with high mortality indicating a need for new therapeutic approaches. We used a k-mer counting approach to identify cancer-specific mRNA transcripts in 3 Ewing’s Family Tumor (EFT) cell lines not found in the normal human transcriptome. Phosphorodiamidate morpholino oligomers targeting six EFT-specific transcripts were evaluated for cytotoxicity in TC-32 and CHLA-10 EFT lines and in HEK293 renal epithelial control cells. Average morpholino efficacy (EC_50_) was 0.66 ± 0.13 in TC-32, 0.25 ± 0.14 in CHLA-10 and 3.07 ± 5.02 µM in HEK293 control cells (ANOVA *p* < 0.01). Synergy was observed for a cocktail of 12 morpholinos at low dose (0.3 µM) in TC-32 cells, but not in CHLA-10 cells. Paired synergy was also observed in both EFT cell lines when the PHGDH pre-mRNA transcript was targeted in combination with XAGE1B or CYP4F22 transcripts. Antagonism was observed when CCND1 was targeted with XAGE1B or CYP4F22, or when IGFBP-2 was targeted with CCND1 or RBM11. This transcriptome profiling approach is highly effective for cancer drug discovery, as it identified new EWS-specific target genes (e.g. CYP4F22, RBM11 and IGBP-2), and predicted effective antisense agents (EC_50_ < 1 µM) that demonstrate both synergy and antagonism in combination therapy.

## INTRODUCTION

Identification of genes or gene segments that are expressed exclusively in tumor cells represents a novel approach to discovery of anti-cancer therapeutics [1]. Most genes are expressed in both tumor and normal tissues so interfering with expression carries potential liability of unwanted toxic effects. A smaller fraction of genes are expressed in normal tissues, but expression is lost in tumors. These normal-specific genes are not likely candidates for therapeutic discovery. An even smaller number of genes or gene segments are expressed in tumors only, and these have the highest potential to be safely targeted with antisense therapeutics. Using transcriptomics [2], we can study the transcriptome of a single tumor, or the sum of all RNA transcripts within an individual cell, tissue, organ or complete organism. This approach captures a snapshot of the target’s functional genome, and allows comparisons across different experimental conditions and time points, elucidating information on gene function, gene regulation and underlying changes to an organism’s biology. There are two primary approaches for transcriptome profiling, including microarrays and RNA-sequencing (RNA-Seq), which employs high-throughput sequencing (HTS) [3, 4].

Here, we annotated the open source RNA-Seq data for each of 26 different normal tissues (testis, colon, spleen, placenta, skin, lung, adipose, stomach, prostate, endometrium, bone marrow, small intestine, cerebral cortex, lymph node, thyroid, kidney, gall bladder, ovary, appendix, adrenal gland, esophagus, salivary gland, heart muscle, duodenum, liver and pancreas) to create a composite of normal (N) gene expression in a healthy human. Next, we annotated the available RNA-Seq data from three, highly-diverse, EFT cell lines (A-673, TC-32 & TTC-446) to create a composite representation of the tumor (T) transcriptome. Central to this process is the computational counting of individual strings (or reads) of sequenced DNA of variable length ‘k’ [5]. Over-represented k-mers in the transcriptome are often of particular biological interest [6, 7]. Here, we counted the abundance of all possible RNA 25-mers in the open source RNA-Seq data for normal and diseased tissues, searching specifically for target genes whose relative expression in a composite of Ewing’s sarcoma is at least 100 times greater than its expression in any normal tissue.

Ewing’s sarcoma (EWS) is a rare disease in which tumors are observed in bone and soft tissue arising from mesodermal and ectodermal tissues [8]. About 90 percent of Ewing’s sarcomas result from a chromosome 11 and 22 translocation (t(11;22) (q24;q12)) which fuses the EWS gene (ch22) to the FLI-1 gene (ch11) [9]. EWS is most common in children with 0.3 cases/million in children aged <3 years old and as high as 4.6 cases/million in children aged 15 to 19 years old. EWS is commonly treated with multidrug chemotherapy consisting of ifosfamide and etoposide as well as surgery (including amputation) and/or radiation [10]. Neoadjuvant therapy may include vincristine, doxorubicin, and cyclophosphamide, and the five-year survival for localized disease is 70 to 80% when treated with chemotherapy but less than 10% if not. The five-year survival for patients with metastatic disease can be as high as 50% but some report 25 to 30% [11–14]. Both CXCR4 and CXCR7 receptors are prognostic of poor outcome with lower expression associated with greater survival (both expressed 5-year survival is >90% but <30% 5-year survival if high expression of both receptors) [15]. Despite some progress in the characterization and treatment of the disease, EWS, which has a high incidence in children, retains a relatively poor survival rate. In this regard, we wish to pursue, new, highly effective anticancer therapy (HEAT) for EWS that exploits improved mechanistic information of the disease based on a deeper understanding of the tumor-specific transcriptome. Here we describe a simple methodology for the rapid characterization of a tumor-specific transcriptome using open-source RNAseq data, and the preliminary evaluation of prominent anti-cancer gene targets using a high-throughput, morpholino-based cytotoxicity assay. While the efficacy, potency and toxicity of single, antisense agents targeting individual, overexpressed genes in primary EWS cell lines was evaluated, a key goal of this study was to also evaluate the potential for combinatorial morpholino regimens to provide synergistic (or antagonistic) antiproliferative effects when administered as a multidrug cocktail that simultaneously targeting several, tumor-specific genes.

## RESULTS

### Personalized antisense medicine via computational transcriptome analysis

**Figure 1:**
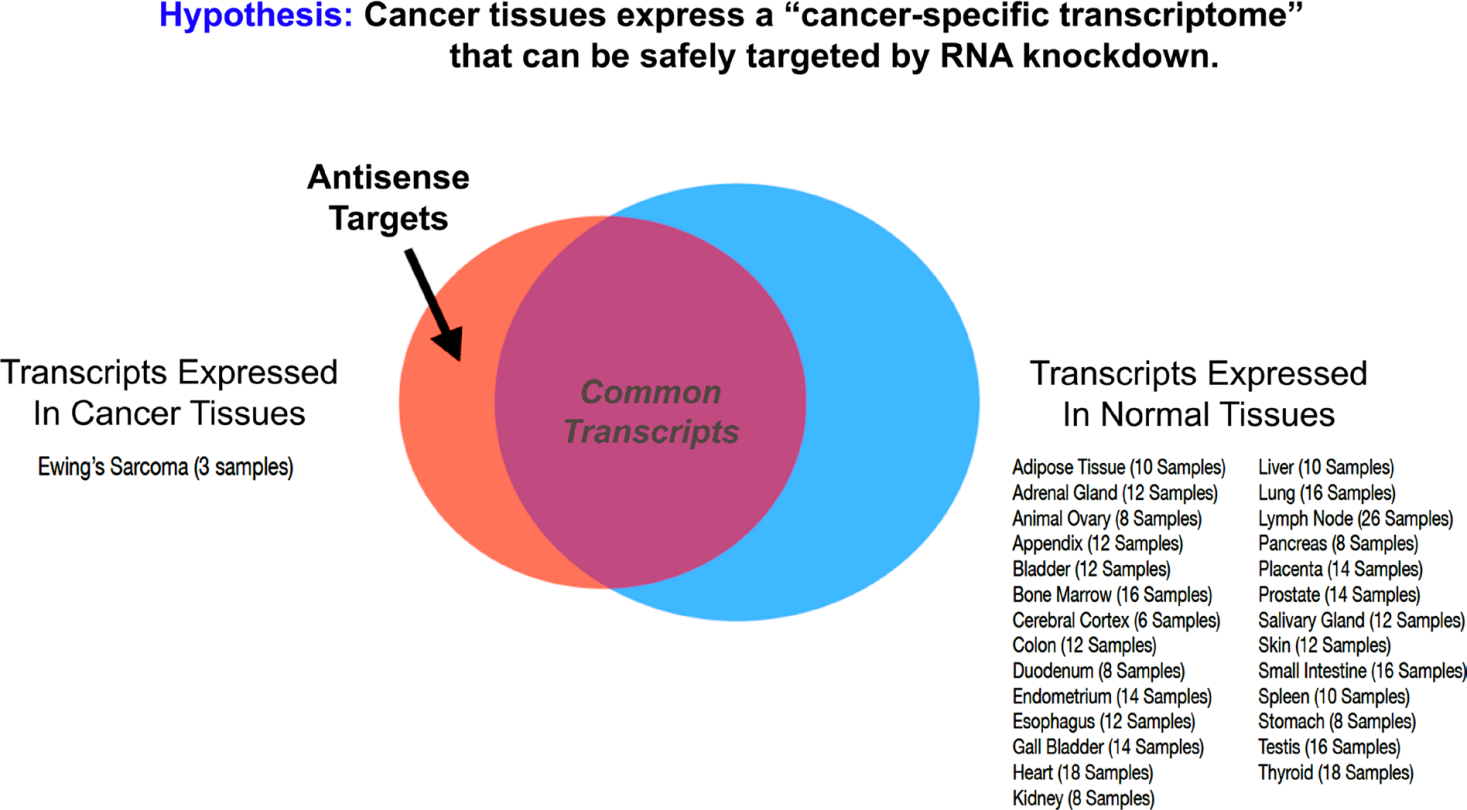
Targeting the cancer-specific transcriptome with antisense oligomer cocktails For any given tumor, a cancer-specific transcriptome exists that only partially overlaps with the normal, healthy transcriptome. Within this cancer-specific pool of mRNA transcripts exists a subset of oncogenic transcripts that convey pro-survival cues via both protein expression and the activity of non-coding RNA. Antisense therapeutics (e.g. morpholinos) that can specifically degrade or modify this subset of RNA transcripts hold unique therapeutic potential as precision-cancer medicines with limited off-target effects in healthy tissues. We annotated a normal, healthy human mRNA transcriptome from a panel of 26 human tissues using 25-mer counts, and compared it to a composite transcriptome profile for Ewing’s Sarcoma (EWS), taken from 3 representative cell lines (TC-32, A673, and TTC-466). Exceptional mRNA transcripts, expressed at approximately 10,000-fold higher levels in EWS cells than in any normal human tissue, were selected for further analysis as potential chemotherapeutic targets using the gene-targeted therapeutic approach highlighted in Table 1 and Figure 2.

To explore the utility of antisense therapeutics in the treatment of human disease, we sought to establish a new computational approach for rapidly predicting new therapeutic regimes. Modulating the expression of a discrete pool of cancer-specific RNA transcripts may hold unique therapeutic potential as a precision anti-cancer medicine. The personalized cancer transcriptome approach avoids off-target sites based on its ability to segregate normal RNA transcripts found in healthy tissues from cancer-specific transcripts found only in tumor cell types (Figure 1). The k-mer-based approach described here relied upon randomized, open source transcriptome data for both normal (26 healthy tissues) and cancerous tissues (3 EWS cell-types).

### K-mer-based transcriptome analysis of Ewing’s sarcoma cell lines

**Figure 2:**
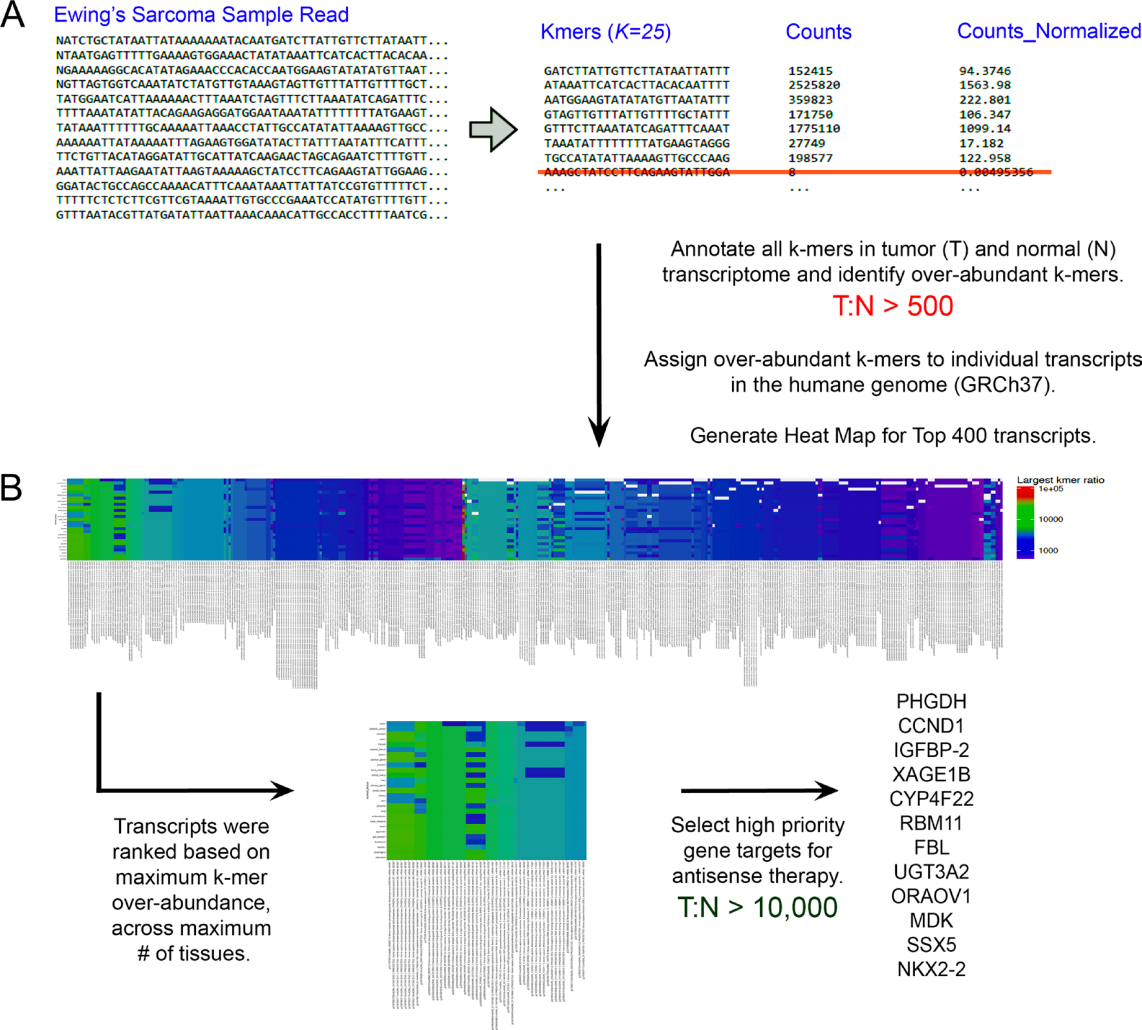
Overview of the k-mer based computational counting approach (**A**) RNA-Seq produces short sequencing reads (∼ 100 bp) from total or poly-A RNA and we can rapidly count all instances of any given ribonucleotide string (length = k) within that dataset. Here we demonstrate the total counts of all, individual 25-mer RNA strand sequences present in an RNA-Seq database for EWS cells, normalized to the total number of sequencing reads for the experiment. 25-mers found to be highly-abundant in EWS cells, with tumor (T) to normal (N) ratios (T:N) greater than 500 were assigned to individual protein coding or non-coding RNA transcripts found in the human genome (GRCh37; hg19). Next, the top 400 transcripts were plotted in a heat map visualizing, for each tissue/transcript combination, the abundance ratio for the selected k-mer across 26 tissues. EWS-specific gene transcripts with k-mer over-abundance levels exceeding 1000-fold over normal cells are colored blue, while those with levels 10,000-fold to 100,000-fold above normal tissues are colored from green to red, respectively. (**B**) Exceptional transcripts identified as having the maximum k-mer over-abundance across the maximum number of tissues were down-selected as high priority leads for antisense inhibition studies. We identified 12 EWS-specific gene targets from regions of the heat map where the T:N ratio approached or exceeded 10,000:1 across all 26 tissues. EFT-specific genes identified include: PHGDH, CCND1, IGFBP-2, XAGE1B/E, CYP4F22, RBM11, FBL, UGT3A2, ORAOV1, MDK, SSX5 and NKX2-2. 6 of the most exceptional genes with unique cellular functions (listed in Table 2) were selected for further analysis using our reverse genetics approach.

An overview of our k-mer-based approach is depicted in Figure 2A, and the details of our down selection process for EFT-specific gene targets is highlighted in Table 1. In summary, we annotated all 25-mer RNA sequence combinations present in the 5.26E+07 reads of RNAseq data for 3 EWS cell lines (A-673, TC-32 & TTC-446) available in the ArrayExpress database (http://www.ebi.ac.uk/arrayexpress) under accession number: E-GEOD-73610 [16, 17]. Next, we annotated all 25-mers present in the 3.74E+09 reads of RNAseq data prepared for the Human Protein Atlas database (www.proteinatlas.org) for normal human tissues accessible from ArrayExpress (accession # E-MTAB-513) [16, 18, 19]. As shown in Table 1, there are 1E+15 possible 25-mer base combinations for the four common ribonucleic acids (A, C, G, U). Using the memory-efficient k-mer counting software Jellyfish [5], we catalogued 5.7E+09 unique 25-mers across both normal and EWS tumor cell lines. We then filtered 1.09E+08 25-mers with a minimum tumor (T) to normal (N) abundance ratio (T:N) of 500:1 and assigned each 25-mer to a corresponding protein coding or non-coding RNA transcript in the human transcriptome (Ensembl GRCh37; hg19 [20]). Next, we visualized the T:N ratio for 400 known transcripts with the highest number of over-representations across normal tissues in a heat map (Figure 2B; high resolution heat map available in Supplementary Figure 1). Ultimately, 6 genes with extreme 25-mer over-abundance (T:N count ratio > 10,000:1) and putative cancer-related mechanisms of action, were down selected as potential targets for antisense-based chemotherapy (Table 2). A 96-well plate cytotoxicity assay, based on the MTT Proliferation assay [21–23], was then developed to compare the ability of antisense oligomers targeting these genes to suppress EFT cell growth at levels comparable to a conventional chemotherapeutic agent, like etoposide.

**Table 1:** *In Silico* selection of precision gene targets in tumor cells

Down selection process	Total k-mers
Computationally annotate all possible 25-mers; (4)^25^; 5′-(NNN…NNN)_25_-3′	10^15^
Catalog and count the occurrence of all 25-mers in both the normal and tumor-specific transcriptome databases.	5.7 × 10^9^
Filter 25-mers with Tumor (T) to Normal (N) expression (T:N) ratios > 500:1.	1.09 × 10^8^
Annotate human transcripts by k-mer overabundance across tissues, ordered by number of tissues with ratios > 500:1, restricted to the top 400 transcripts.	400
Select lead therapeutic targets; Priority is given to targets with T:N ratios > 10,000:1 across the greatest number of normal tissues; Design morpholinos to target exceptional tumor-specific genes to validate their role in the disease-state.	12

**Table 2:** Morpholino oligomers evaluated

Gene name	Activity	Name	Oligomer sequence
Scramble Control Oligomer	N/A	Scr	CCTCTTACCTCAGTTACAATTTATA
X antigen family member 1 (XAGE1B)	Enhances anti-apoptotic effect of TNF-α	XAGE1B	CTGTGTGGTTCTGTGTTTGT
Cyclin D1 (CCND1)	Regulates cell cycle progression	CCND1	CTCGGCTCTCGCTTCTGCTG
RNA binding motif protein 11 (RBM11)	Tissue-specific regulator of gene splicing	RBM11	AATGAAGTAGGAGCTGAGACCCC
Cytochrome P450 family 4 subfamily F member 22 (CYP4F22)	Regulates acyl-ceramide metabolism	CYP4F22-1 CYP4F22-2 CYP4F22-3	TCTGTGATGGGCAGCATCCT TTGGGTTCACTGTCTTCTTCCTTGC TGCCATGCACAAGACGATGACCCA
Phosphoglycerate dehydrogenase (PHGDH)	Plays essential role in serine biosynthesis	PHGDH-1 PHGDH-2	CGCTGTGAGTAGAAGTACCTAAGCC AAGCCGCAGGCACATCATTGCTTAC
Insulin like growth factor binding protein 2 (IGFBP-2)	Enhance or attenuate pro-survival, IGF signaling	IGFBP2-1 IGFBP2-2 IGFBP2-3 IGFBP2-4	CTCCTCCGCTTCTTCCTCCT TGATGTCTGTCCAACAAGCGTCCAT TGCTCAGTGACCTTCTCCCGGAAC TGGTTCTCCACCAGGCCTCCTTC

### Benchmark etoposide cytotoxicity in Ewing’s family of tumors

Etoposide, a DNA topoisomerase II inhibitor, has demonstrated potent activity against a large number of tumor types including soft tissue sarcomas such as Ewing’s, particularly in combination with DNA alkylating agents (such as ifosfamide) [24]. To compare the cytotoxicity of our k-mer derived oligomers shown in Table 2 to a well-characterized therapeutic agent, we pre-validated the etoposide sensitivity of a panel of EFT cell lines (TC-32, TC-71, CHLA-9 and CHLA-10) obtained from the Children’s Oncology Group (COG) Cell Culture and Xenograft Repository [25–28], using 96-well, morpholino-based cytotoxicity assay (see methods). Collectively, these EFTcell lines have a reported EC_50_ value for etoposide of approximately 0.1–0.12 ng/mL (or ∼0.2 nM), and EC_90_ values range from ∼ 1 to 10 ng/mL (2–20 nM) [27, 28]. We employed a conservative dose of etoposide (5 doses, 0.1–10 nM) to establish a 24 hour benchmark level of cytotoxicity for each EFT cell lines tested. As shown in Table 3, the CHLA-10 cell line was most sensitive to etoposide in our assay (EC_50_ = 0.32 µM); followed by CHLA-9 cells (EC_50_ = 0.42 µM), TC-71 cells (EC_50_ = 0.56 µM) and TC-32 cells (EC_50_ = 0.63 µM). All four EFT cell lines were significantly more sensitive to etoposide than a HEK293 emybryonic kidney cell control (EC_50_ = 0.72 nM; see Table 3). Based on this range of sensitivities, we selected TC-32 and CHLA-10 cells for further comparison against our panel of 12 morpholino oligomers targeting 6 exceptional EFT-specific genes.

**Table 3:** Benchmark etoposide cytotoxicity in EWS cell lines

Cell line	Efficacy (EC_50_) *µM*	Sensitivity_24 hrs_ % dead cells per nM morpholino (*r*^2^)	Fraction dead cells at 24 hours (FA_24 hs_)^†^
*HEK293 (control)*	*0.73*	*0.068 (0.895)*	*0.16 ± 0.01*
TC-32	0.64	0.078 (0.900)	0.26 ± 0.05^*^
CHLA-9	0.43	0.117 (0.889)	0.32 ± 0.03^*^
CHLA-10	0.32	0.156 (0.915)	0.41 ± 0.05^*^

^†^Fraction Dead Cells at 24 hours (FA_24 hrs_) represents the activity of the agent and is calculated for individual cell lines using the fraction of cells lost among 5 biological replicates at the most potent concentration of etoposide, corrected to the average fraction of cells lost using a DMSO vehicle control only; All replicates were measured on the same 96-well tray and were seeded with an identical number of starting cells, 24 hours prior to etoposide treatment (see methods).

^*^p < 0.05, based on paired student *t*-test comparing mean corrected activity (FA_24 hrs_) at 24 hours.

### Single agent activity for morpholinos targeting EFT cells and an unrelated HEK293 cell control

The cytotoxic activity, efficacy and sensitivity of single morpholino agents listed in Table 2 were established in two, primary EFT cell lines (TC-32 and CHLA-10) using our 96-well cell viability assay (see methods). Morpholino activity was evaluated by measuring the fraction of dead cells at 24 hours after treatment (FA_24hs_) for 5 doses of total oligomer ranging from 0.03–3 µM. An ANOVA analysis of peak morpholino activity (e.g. FA_24hs_ for the most potent dose) was performed among HEK293, TC-32 and CHLA-10 cells (Table 4). At the 0.3 µM dose no morpholinos had a statistically significant effect on HEK293 control cell growth. Cytotoxicity of single agents was significantly different in CHLA-10 cells at the same 0.3 µM, dose and in TC-32 cells at the 3.0 µM dose, where peak activity was observed. Using a paired student *t*-test we confirmed that 11 of the 12 morpholinos were more cytotoxic in EFT cell lines than the HEK293 control, with only the CCND1-targeted morpholino being insignificant for TC-32 cell-specific cytotoxicity (Table 4).

**Table 4:** ANOVA analysis of single agent activity^#^ or fraction cells affected at 24 hours

Cell type	HEK-293	TC-32	CHLA-10	*T*-Test	
Antisense Target	0.3 µM	3.0 µM^†^	0.3 µM	HEK vs TC-32	HEK vs CHLA-10
XAGE1E	−0.09 ± 0.06^*^	0.19 ± 0.10	0.59 ± 0.18	0.0001	0.0000
CCND1	0.03 ± 0.04	0.05 ± 0.04	0.45 ± 0.07	0.2932	0.0000
RBM11	−0.15 ± 0.05	0.10 ± 0.04	0.37 ± 0.05	0.0000	0.0000
CYP4F22-1	−0.02 ± 0.02	0.13 ± 0.05	0.10 ± 0.07	0.0000	0.0076
CYP4F22-2	−0.02 ± 0.09	0.17 ± 0.07	0.18 ± 0.07	0.0016	0.0023
CYP4F22-3	−0.07 ± 0.04	0.24 ± 0.10	0.06 ± 0.04	0.0000	0.0004
PHGDH-1	0.08 ± 0.08	0.22 ± 0.18	0.34 ± 0.22	0.0337	0.0341
PHGDH-2	0.09 ± 0.03	0.20 ± 0.08	0.30 ± 0.07	0.0006	0.0002
IGFBP2-1	0.03 ±0.07	0.18 ±0.05	0.28 ± 0.11	0.0027	0.0014
IGFBP2-2	0.02 ± 0.02	0.30 ± 0.21	0.25 ± 0.08	0.0007	0.0004
IGFBP2-3	0.03 ± 0.07	0.14 ± 0.04	0.50 ± 0.15	0.0151	0.0002
IGFBP2-4	0.02 ± 0.02	0.19 ± 0.05	0.42 ± 0.16	0.0000	0.0015
ANOVA	F = 3.1; ns	F = 5.6; *p* < 0.01	F = 9.3; *p* < 0.01		

^**#**^Fraction Dead Cells at 24 hours (FA_24 hs_); FA_24 hrs_ was calculated for single agents using the fraction of cells lost among 5 replicate samples, corrected to the average fraction of cells lost when treated with the highest a scramble control morpholino (for doses ranging from 0.1 to 3 µM) + 10 µM Endo-Porter; Replicates were and controls were measured in the same 96-well seeded with the identical number of healthy cells 24 hours prior to treatment.

^*^HEK293 cells show no significant fraction affected cells at 0.3 µM for any antisense agent.

^†^TC-32 cells were resistant to Endo-Porter based morpholino uptake and showed peak cytotoxicity at the 3.0 µM dose delivered with passive uptake. CHLA-10 cells showed peak cytotoxicity at a 0.3 µM dose with Endo-Porter.

Using the same data set, we also computed the average efficacy (EC_50_) and sensitivity (% dead cells per nM morpholino) for each morpholinos tested in all 3 cell lines (Table 5). The average efficacy of single agents in HEK293 control cells was 3.1 ± 5.0 µM, with a broad range for individual agents varying from 0.46 to 16.7 µM. The sensitivity of individual agents in HEK293 cells, as measured by the slope of the oligomer concentration versus FA_24hrs_ line, was 0.05 ± 0.05% dead cells per nM morpholino, not significantly different from zero. The efficacy for morpholinos targeting XAGE1E (EC_50_ = 0.73 µM), CCND1 (EC_50_ = 0.49 µM), RBM11 (EC_50_ = 0.65 µM) and CYP4F22 (EC_50_ = 0.45–0.51 µM) were not substantially greater than activity observed in TC-32 cells or CHLA-10 cells (Table 5). Markedly reduced efficacy and sensitivity was observed in HEK293 cells for morpholinos targeting PHGDH and IGFBP-2 transcripts. Furthermore, the observation that multiple agents targeting a single gene are consistently active, such as the three targeting CYP4F22, or consistently inactive, such as the 2 oligomers targeting PDGH and the 4 oligomers targeting IGFBP, tends to confirm the capability of morpholinos to probe a target gene’s role in supporting cell growth. Results in HEK293 cells indicated that k-mer-based transcriptome profiling can identify active targets that are both specific (PHGDH and IGFBP-2) and non-specific (XAGE 1E, CCND1, RBM11 and CYP4F22) for the targeted cancer, as well as targets that are relatively inactive (PHGDH and IGFBP-2) in unrelated cell lines.

**Table 5:** Single agent efficacy^†^ and sensitivity^‡^ at 24 hours

Cell type	HEK293 cells	TC-32 cells	CHLA-10 cells
Antisense target	*Efficacy* (EC50) µM; (Sensitivity = % dead Cells per nM morpholino; *r*^2^)		
XAGE1E	*0.73*; (0.068; *r*^2^ = 0.88)	*0.65*; (0.076; *r*^2^ = 0.92)	*0.12*; (0.408; *r*^2^ = 1.00)
CCND1	*0.49*; (0.101; *r*^2^ = 0.87)	*0.74*; (0.067; *r*^2^ = 0.96)	*0.16*; (0.312; *r*^2^ = 0.99)
RBM11	*0.65*; (0.076; *r*^2^ = 0.83)	*0.85*; (0.059; *r*^2^ = 0.90)	*0.14*; (0.364; *r*^2^ = 1.00)
CYP4F22-1	*0.51*; (0.098; *r*^2^ = 0.76)	*0.67*; (0.074; *r*^2^ = 0.92)	*0.34*; (0.145; *r*^2^ = 1.00)
CYP4F22-2	*0.45*; (0.109; *r*^2^ = 0.95)	*0.57*; (0.087; *r*^2^ = 0.99)	*0.35*; (0.144; *r*^2^ = 0.88)
CYP4F22-3	*0.50*; (0.099; *r*^2^ = 0.98)	*0.49*; (0.100; *r*^2^ = 0.94)	*0.56*; (0.090; *r*^2^ = 0.74)
PHGDH-1	*1.31*; (0.038; *r*^2^ = 0.70)	*0.66*; (0.075; *r*^2^ = 0.95)	*0.11*; (0.464; *r*^2^ = 0.99)
PHGDH-2	*1.84*; (0.027; *r*^2^ = 0.79)	*0.75*; (0.067; *r*^2^ = 0.91)	*0.36*; (0.137; *r*^2^ = 0.81)
IGFBP2-1	*9.98*; (0.005; *r*^2^ = 0.04)	*0.62*; (0.080; *r*^2^ = 0.91)	*0.29*; (0.170; *r*^2^ = 0.61)
IGFBP2-2	*1.85*; (–0.027; *r*^2^ = 0.93)	*0.41*; (0.120; *r*^2^ = 0.92)	*0.27*; (0.185; *r*^2^ = 0.91)
IGFBP2-3	*16.6*; (–0.003; *r*^2^ = 0.04)	*0.88*; (0.056; *r*^2^ = 0.96)	*0.12*; (0.405; *r*^2^ = 0.81)
IGFBP2-4	*1.85*; (–0.027; *r*^2^ = 0.93)	*0.67*; (0.074; *r*^2^ = 0.98)	*0.21*; (0.235; *r*^2^ = 0.79)
	**Average ± SD**		
*(EC*_*50*_*)*	3.07 **±** 5.02 µM	0*.*66 ± 0.13 µM	0*.*25 ± 0.14 µM
*% Dead Cells per nM*	0.05 ± 0.05	0.078 ± 0.002	0.25 ± 0.13

^**†**^Efficacy at 24 hours represents or (EC_50_) was determined by linear regression analysis of the line plotting fraction cells affected at 24 hours (FA_24hs_) versus the concentration of morpholino (nM). EC50 was calculated using the formula y = mx + c where y = 50; *r*^2^ values for the regression line are listed in the table with the sensitivity data.

^‡^Sensitivty at 24 hours or % Dead Cells per nM morpholino was calculated using a linear regression analysis of the line plotting fraction cells affected at 24 hours (FA_24hs_) versus the concentration of morpholino (nM). Sensitivity = the slope (m) in the equation y = mx+c. r^2^ values for the regression line are listed in the table with the sensitivity data.

The average efficacy (EC_50_) for all 12 morpholinos in TC-32 cells was 0.67 ± 0.13 µM on the order of five times lower than the unrelated HEK293 cell line (0.67 versus 3.07 µM). The average sensitivity of 0.078 ± 0.002 percent dead cells per nM oligomer also revealed a greater response to antisense inhibition compared to the HEK293 cell line (0.078 ± 0.002 versus 0.05 ± 0.05). For TC-32 cells, the EC_50_ range was limited from 0.42 to 0.89. Individual morpholinos targeting the IGFBP-2 (variant 2) and CYP4F22 (variant 3) genes were more effective than the average single agent with EC_50_ values of 0.42 µM and 0.50 µM and sensitivity values of 0.120 and 0.100 percent dead cells per nM oligomer, respectively (Table 5). Two additional morpholinos targeting CYP4F22, and 3 additional morpholinos targeting IGFBP-2 were also effective as they suppressed cellular proliferation (12–19%) compared to a scrambled control oligomer or a DMSO vehicle control which showed ≤4% cytotoxicity across all experimental replicates. Morpholinos targeting XAGE1, CCND1, RBM11 and PHGDGH (2) were generally less effective, but still showed a consistent ability to suppress TC-32 proliferation (at rates ∼4-20-fold above control levels). Morpholinos targeting IGFBP-2 (24%) and CYP4F22 (23%) were able to suppress TC-32 proliferation at rates comparable to etoposide at 24 hours (26%), far exceeding the suppression seen for vehicle (DMSO) or scrambled oligomer controls.

Next, we established the efficacy and sensitivity of our morpholinos in the CHLA-10 EFT cell line, which showed the highest sensitivity to etoposide at 24 hrs (Table 3; EC_50_ = 0.32 µM; 41% fraction dead). The average efficacy (EC_50_) for all 12 morpholinos in CHLA-10 cells was 0.25 ± 0.14 µM which is more than 2.5-fold lower than for the TC-32 cell line, and over 12-fold lower than the unrelated HEK293 cell line (Table 5). The average sensitivity of 0.254 ± 0.013 percent dead cells per nM oligomer also reveals a much greater response to the morpholinos in CHLA-10 cells compared to either the TC-32 or HEK293 cell lines (0.25 versus 0.08 and 0.05, respectively). The efficacy for single agents ranged from 0.11 to 0.56 µM in CHLA-10 cells, suggesting once again that the computational transcriptome analysis successfully identified important EFT targets. The average cytotoxicity of the morpholino scrambled control was 5.9%, and did not exceed 13% for any replicate controls in CHLA-10 cells, confirming morpholino sequence specificity. Individual morpholinos targeting PHGDH-1, IGFBP-2 (variant 3), XAGE1, and RBM11 genes were nearly twice as effective as the average single agent in CHLA-10 cells, with EC_50_ values ranging from 0.11–0.14 µM, and sensitivity values of 0.36–0.46 percent dead cells per nM oligomer, respectively (Table 5). These findings demonstrate the potential of this method to identify cancer-specific drug targets and gene-specific morpholinos with dose dependent cytotoxic effects comparable to etoposide at 24 hrs, and EC_50_ values on average, well below 1.0 µM.

### Assessing morpholino cocktail synergy in TC-32 cells

**Figure 3:**
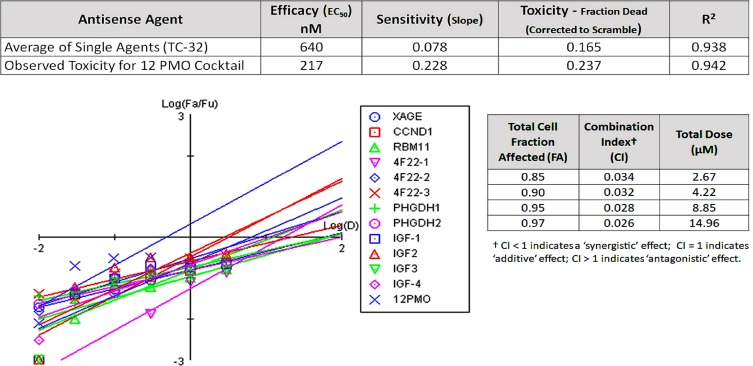
Assessing the synergistic potential of antisense cocktails to suppress EFT proliferation The goal of this project was to identify a panel of gene targets highly-expressed in Ewing’s Sarcoma malignancies only, for use in a combinatorial antisense therapy using morpholinos. After screening the dose-responsiveness of single agents to suppress TC-32 cell growth, we conducted a comparison screen that utilized a cocktail of all 12 morpholinos, over the same concentration range. The average single agent displayed an average efficacy (EC_50_) of 640 nM, killing roughly 17% of TC-32 cells at 24 hours exposure. In contrast, the cocktail of 12 oligomers showed greater sensitivity, with an EC_50_ of 217 nM that resulted in 24% cell death at 24 hours. CompuSyn results comparing the log dose (D) to the log fraction affected/fraction unaffected (Fa/Fu) reveal the enhanced cell killing at log doses below 0. CompuSyn analysis of synergy for the 12 morpholinos revealed a combination index (CI) well below zero (0.026–0.034), indicating strong synergy of the cocktail. At higher doses of the cocktail (1–3 µM total oligomer), suppression of TC-32 cell growth was negligible (not shown), and thus synergy was not evident beyond the intermediate dose of the cocktail (0.3 µM), hinting at the potential for enhanced antagonism among agents, at doses of mixed oligomer exceeding 1 µM.

TC-32 cells displayed the greatest resilience to both etoposide and individual morpholino agents using passive uptake and Endo-Porter delivery methods. However, we also explored the concentration dependent TC-32 cell sensitivity (% dead cells per nM morpholino) of a combination therapy using all 12 morpholinos, where each compound in the cocktail represented 1/12th of the total oligomer concentration between 0.01–0.3 µM. As shown in Figure 3, the cocktail efficacy was nearly twice as potent as the best single agent (EC_50_ = 0.22 µM vs. 0.41 µM for IGFBP2-2), and almost three times as potent as the average singe agent (EC_50_ = 0.64 µM), at concentrations between 0.1 and 0.3 µM total oligomer. The 12 morpholino cocktail was 140% more effective at killing TC-32 cells at 24 hours than the average single agent (Figure 3; Fraction dead = 0.24 vs. 0.17) and suppressed TC-32 cell proliferation on par with etoposide at 24 hours (24% versus 26%, respectively).

A CompuSyn analysis of synergy [29], for the 12 morpholino cocktail experiment, revealed strong synergy for individual agents in TC-32 cells with combination index (Cl) values ranging from 0.026–0.034 for total cell fraction affected (FA) values ranging from 0.85–0.97 [29]. For these algorithms, Cl values below 1 indicate synergistic effects, Cl values equal to 1 indicate additive effects, and Cl values greater than 1 indicate antagonism (Figure 3).

### Assessing morpholino synergy in TC-32, CHLA-10 cells

**Figure 4:**
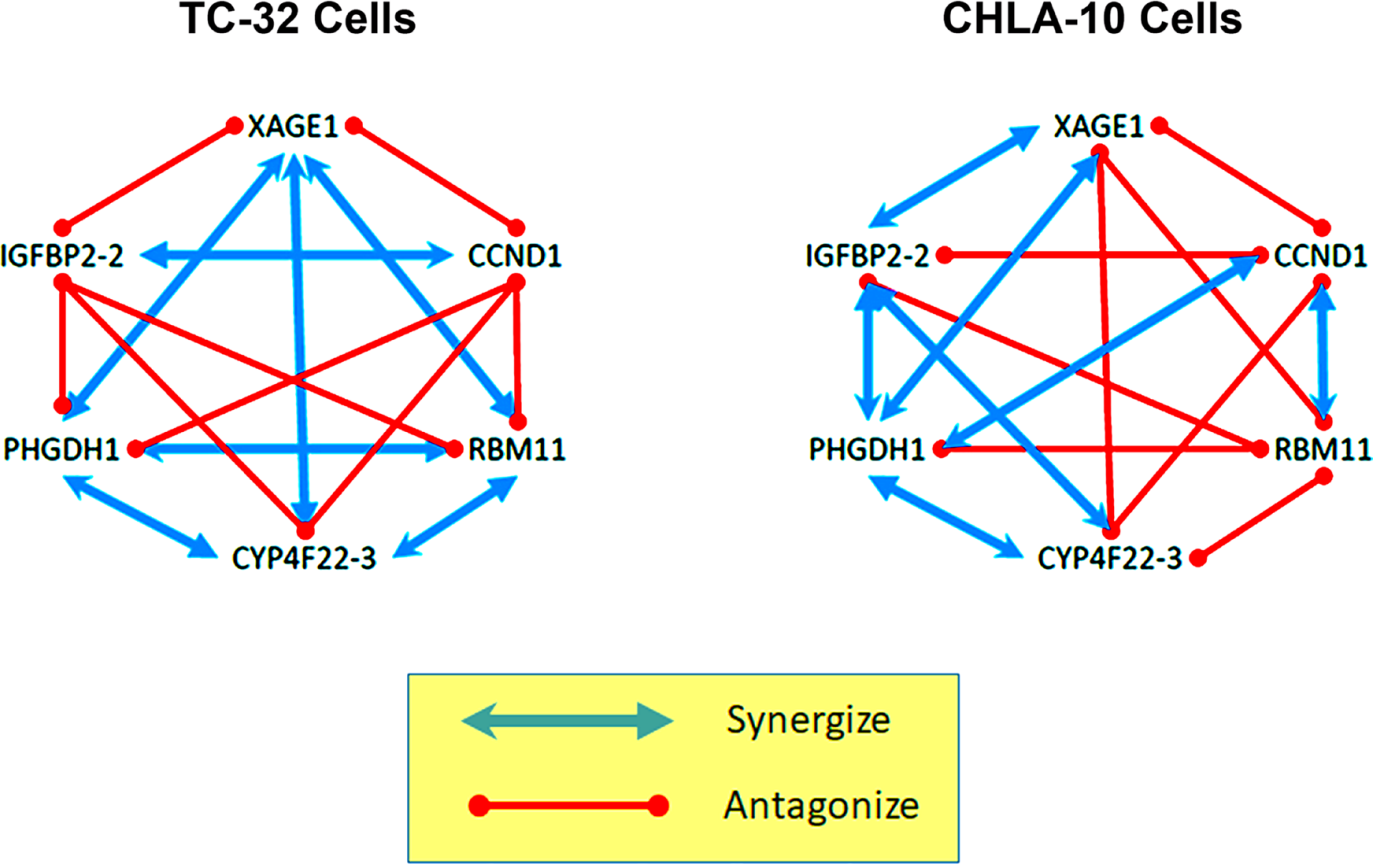
Tumor-specific gene interaction networks revealed by the reverse genetics approach An oncogene can be selectively-expressed or over-expressed in a tumor cell, but deciphering the role it plays in promoting cell survival is often complicated by competing or compensatory gene pathways that can modify their effects. Antisense therapeutics allow multiple oncogenes to be targeted, simultaneously, using a cocktail of agents, however we found that at high doses some morpholino cocktails are less efficient than single agents at suppressing EFT cell growth. To unravel the underlying complexity of this phenomenon, we conducted a screen of individual oligomer pairs, and compared their paired toxicity, with results for single agents using the CompuSyn program. We discovered two, unique gene-specific interactomes operating within the highly-related, but distinct EFT cell lines (TC-32 and CHLA-10). Combinatorial pairs that included agents targeting IGFBP-2 or CCND1 were most likely to promote antagonism in TC-32 cells, while pairs targeting RBM11, were most antagonistic in the CHLA-10 cell line. These complex patterns of interactivity hint at the underlying biological heterogeneity that defines individual tumor types, demonstrating both the challenge and importance of personalized approaches of cancer medicine.

To address the ability of individual morpholinos to antagonize or synergize the bioactivity of the other single agents found in the cocktail formulation, we compared the ability of each single agent to alter the potency of the related oligomers. To expedite this analysis we reduced the total number of agents to screen to 6, by selecting the most potent single agent, on average, per overexpressed target gene (XAGE1, CCND1, RMB11, CYP4F22-3, PHDGH-1, and IGFBP2-2). Each combination of two morpholinos was screened at 3 concentrations (0.1, 0.3 and 1.0 µM total morpholino) and the resulting cell viability data was analyzed using the CompuSyn software. Synergy (CI < 1) was observed in TC-32 cells when morpholinos targeting XAGE1 were paired with agents targeting RBM11, CYP4F22-3, and PHDGH-1; when morpholinos targeting CCND1 were paired with those targeting IGFBP2-2; when morpholinos targeting RBM11 were paired with those targeting CYP4F22-3 and PHGDH-1; or when morpholinos targeting CYP4F22-3 were paired with those targeting PHGDH-1 (Table 6 above the diagonal; Figure 4). Additive (CI = 1) was observed in TC-32 cells when morpholinos targeting CCND1 were paired with those targeting PHGDH-1. Antagonism was observed in TC-32 cells when morpholinos targeting XAGE-1 were paired with those targeting CCND1 and IGFBP2-2; when morpholinos targeting CCND1 were paired with those targeting RBM11 and CYP4F22-3; when morpholinos targeting RBM11 were paired with IGFBP2-2; when morpholinos targeting CYP4F22-3 were paired with those targeting IGFBP-2-3; and when morpholinos targeting PHGDH-3 were paired with those targeting IGFBP2-2.

**Table 6:** CompuSyn combination index^a^ at ED_50_ values

	XAGE1	CCND1	RBM11	CYP4F22	PHGDH	IGFBP-2
**XAGE1**		1.1e66	**4.5e-17**	**0.15**	**0.000047**	210
**CCND1**	*38.0*		5100	320	1.10	**0.00000051**
**RBM11**	*8500*	**0.0044**		**0.00000022**	**0.74**	6.0e17
**CYP4F22**	*5400*	*70.0*	*8.9*		**0.20**	5900
**PHGDH**	**0.10**	**0.00074**	*7.4e8*	**0.0079**		5.7
**IGFBP-2**	**0.000025**	*620*	*9.1*	**0.082**	**0.011**	

^a^CompuSyn-derived combination Index (CI); Numbers in *italics* below the diagonal represent CHLA-10 cell observations. Numbers above the diagonal represent TC-32 cell observations. CI values of 1 indicate additive effect; CI > 1 indicates antagonism in the combination; CI < 1 indicates synergism in the combination.

Each combination of single agents was also evaluated in CHLA-10 cells at 3 concentrations (0.1, 0.3 and 1.0 µM total morpholino) and resulting data was analyzed using the CompuSyn software. Synergistic activity (CI < 1) was observed in CHLA-10 cells when morpholinos targeting XAGE1 were paired with agents targeting PHDGH-1 and IGFBP2-2; when morpholinos targeting CCND1 were paired with agents targeting RBM11 and PHGDH-3; when morpholinos targeting CYP4F22-3 were paired with agents targeting PHGDH-3 and IFGBP-2-2; and when morpholinos targeting PHGDH-3 were paired with agents targeting IFGBP2-2 (Table 6 below the diagonal; Figure 4). Additive activity (CI = 1) was not observed in CHLA-10 cells. Antagonistic activity (CI > 1) was observed in CHLA-10 cells when single agents targeting XAGE-1 were paired with morpholinos targeting CCND1, RBM11 or CYP4F22-3; when single agents targeting CCND1 were paired with morpholinos targeting CYP4F22-3 or IGFBP2-2; and when single agents targeting RBM11 were paired with those targeting CYP4F22-3, PHGDH-3, or IGFBP2-2.

Using CompuSyn analysis we found that each pairing of morpholinos can promote a complex combinatorial effect that ranges from synergistic to additive to antagonistic, in an EFT cell-line-specific manner. Most notable for the TC-32 cells, morpholinos targeting CCND1 only synergized with those targeting IGFBP2-2. The single agent targeting IGFBP2-2 was equally antagonistic to all the other morpholinos tested, except for the one targeting PHGDH-1, which in combination, gave a mild additive effect (CI = 5.7). These trends were not recapitulated in the CHLA-10 cell line which showed greater synergistic activity when CCND1 was targeted in combination with either RBM11 or PHDGH1-1. Combinations including a morpholino targeting IGFBP2-2 were also more synergistic in the CHLA-10 cells, with enhanced cellular toxicity observed in mixtures including single agents targeting either XAGE1, CYP4F22-3 or PHDGH-1. Morpholino combinations that included oligomers targeting PHDGH-1 and either XAGE1 or CYP4F22-3 showed the most consistent synergistic effects across both EFT cell lines.

## DISCUSSION

The initial goal of this project was to assess whether open source transcriptome profiling could rapidly elucidate a potent, antisense therapeutic regimen for cancer with comparable or superior efficacy to conventional chemotherapeutic agents. With RNAseq technology being increasingly used to characterize the molecular basis of disease, there are now large repositories of transcriptome data available to the public, via sites like the ArrayExpress database (http://www.ebi.ac.uk/arrayexpress), which can be computationally-mined to unravel the biomedical mysteries of both genetic and environmental disorders, and to guide rational drug design [16]. Our group has hypothesized that for diseases like cancer, there is a disease specific-transcriptome that promotes tumor survival that is essentially exclusive to the patient’s healthy transcriptome, and that the cancer-specific pool of transcripts can be safely targeted by antisense therapeutic intervention with no off-target effects in normal tissues.

To test this concept, we set out to characterize the complexity of the tumor-specific transcriptome for the Ewing’s family of Tumors (EFT), which include EWS and peripheral primitive neuroectodermal tumors (PNET). EWS is the second most common form of bone cancer affecting children, and while there is a high survival rate for localized tumors (∼70%), the outcome for patients with metastatic or recurrent bone tumors remains poor (∼20% survival at 5 years) [8–11, 27]. The standard chemotherapy for localized EFT includes vincristine, ifosfamide, doxorubicin and etoposide (VIDE) in Europe, or vincristine, doxorubicin, cyclophosphamide, ifosfamide and etoposide (VDC-IE) in North America [12]. Each of these therapies is associated with off-target effects that limit their use and efficacy, and attempts to intensify the therapeutic effects of these agents has been linked to a high incidence of secondary malignancies [13, 14]. Our goal was to identify new therapeutic gene targets for EWS, and to establish a new, antisense-based therapeutic regimen for patients with metastatic or recurrent EWS, based only on a computational meta-analysis of open source, whole transcriptome data for the disease.

To accomplish this goal we developed a k-mer based counting strategy for cataloging all mRNA transcripts identified in 3 prototype EFT cell lines (A-673, TC-32 & TTC-446) and 26 healthy human tissues taken from the Human Protein Atlas [19]. The RNA-Seq data we selected to represent the EWS transcriptome was originally developed by Town *et al.* [17], and it represents a nice diversity of disease-related phenotypes, encapsulating both peridiploid (A-673, TC-32) and peritriploid (TTC-446) karyotypes, and both the common t(11;22)(q24;q12) chromosomal translocation that produces the EWS/FLI-1 fusion protein (e.g. A-673 and TC-32 cells) as well as the more rarified t(21;22)(q22;q12) translocation that yields the EWS/ERG fusion protein (e.g. TTC-446 cells) [30]. Using cloud computing, we then cataloged the frequency of every possible 25-mer sequence in the transcriptomes of the 26 normal tissues and the 3 EWS cell lines, and we used this database to re-construct a list of gene fragments found highly overexpressed (x > 1000:1) only in EFT cells. A list of 12 genes containing k-mers over-represented in EFT cells at ratios exceeding 10,000:1 compared to normal tissues were down-selected as putative candidates for antisense therapy, and a final list of 6 gene targets was selected based on their putative relevance to EFT pathology or known mechanism of action in cancer.

**Figure 5:**
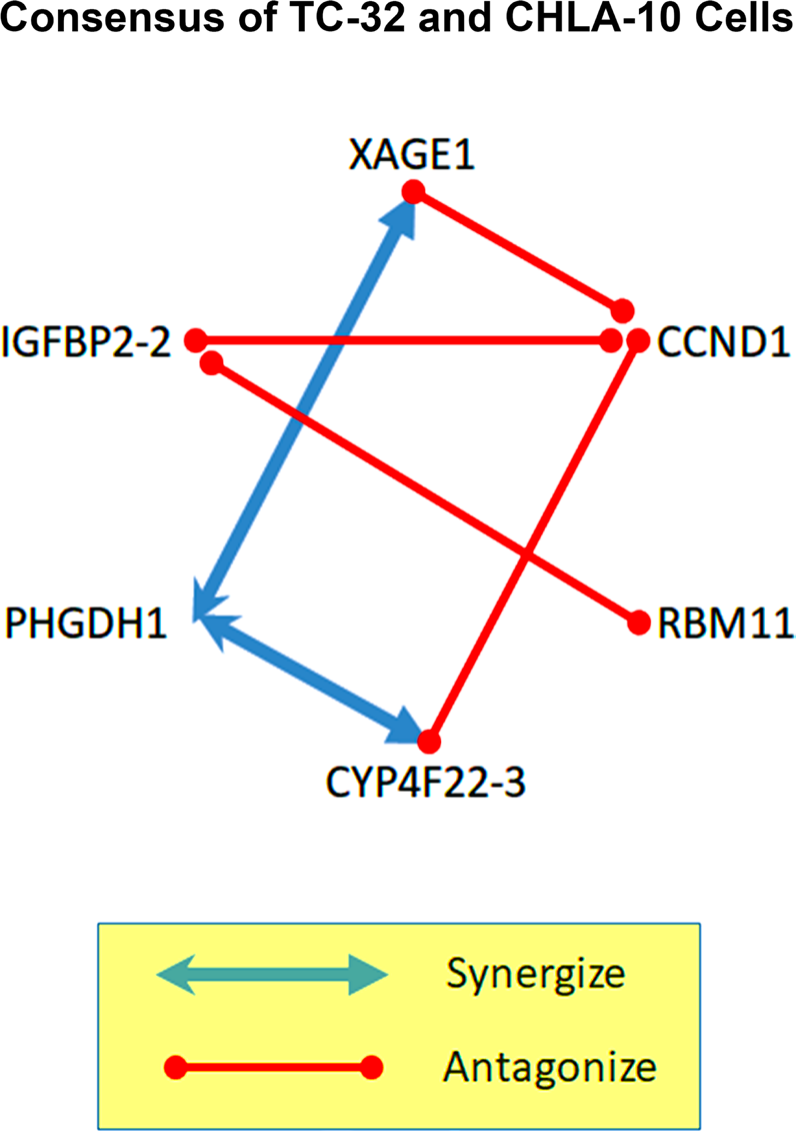
Consensus gene interactome for Ewing’s family tumors While individual single agents displayed a range of anti-tumor activities in EFT cell lines, we were surprised to learn that cocktails targeting all 6 genes simultaneously were less effective than many single agents when administered at high combined doses (> 1 µM). The complete interactome of agents tested in two EFT cell lines is shown in Figure 4. Here we demonstrate the consensus interactome of gene targets operating across both cell lines, one representing an untreated EFT tumor form (TC-32) and the other an aggressive, drug resistant phenotype (CHLA-10). Consensus results indicate that combinatorial therapies targeting PHGDH1 and either CYP4F22 or XAGE1 have an enhanced potential for synergy among diverse EFT tumors. In contrast, combinatorial approaches that target IGFBP-2 or CCND1 in combination are more likely to promote antagonism, particularly when paired with each other.

Our initial screen of a 12 morpholinos targeting 6 exemplary genes (XAGE1, CCND1, RBM11, CYP4F22, PHDGH1 and IGFBP-2) over-represented in a prototype EFT transcriptome, demonstrated cell line-specific sensitivity between the TC-32 and CHLA-10 cells, which both represent clinically-relevant EFT genotypes that were validated by STR analysis prior to use. When administered in combination or as a component of the morpholino cocktail, the activity of the some single agents was antagonized, as cocktails showed strong synergy at low doses, but limited synergy and cytotoxicity at doses above 1 µM compared to single agents (Figure 3). These results demonstrate the challenge of developing a broad spectrum agent to treat diverse Ewing’s family tumors, or combinatorial therapeutic regimens with complex mechanisms of action that can conflict with the underlying tumoristatic or tumoricidal properties of single agents in highly unpredictable ways. For example, single agent morpholinos targeting the transcripts of CYP4F22 or IGFBP2 genes, administered from 0.1–3 µM, were capable of suppressing TC-32 cell growth approximately 24% at 24 hours, which was comparable to the cytotoxicity observed for etoposide at 24 hours (26% cell death). However, when administered in combination, morpholinos targeting CYP4F22-3 and IGFBP2-2 revealed strong antagonism (CI > 10), and reduced suppression of TC-32 proliferation (Figure 4; Table 6). In contrast, a combination of morpholinos targeting both CYP4F22-3 and PHDGH-1 showed a synergistic enhancement in cytotoxicity compared to single agents, in both TC-32 cells and CHLA-10 cells. As shown in Figure 5, we observed cell-specific diversity in the ability of individual morpholino pairs to synergize or antagonize with each other. Therefore, shotgun approaches where multiple tumor-specific genes are targeted simultaneously with combinatorial therapeutics should be approached with caution, as they may fail to achieve the desired efficacy, due to cell-specific differences in gene expression, drug uptake and compensatory biological mechanisms underlying redundant cell functions.

The goal of this study was to validate the existence of a robust, tumor-specific transcriptome operating in EFT cells, and to identify new therapeutic targets, not previously considered for treating EWS. In this regard we identified 9 morpholinos targeting EFT-specific genes (i.e. XAGE1, RBM11, CCND1, PHDGH and IGFBP-2) that suppressed CHLA-10 cell growth at levels superior to etoposide at 24 hours. This result is significant because the EWS-specific transcriptome used for k-mer profiling did not include RNAseq data from CHLA-10 cells, which represent a metastatic EFT lesion of the throat, with non-functional P53 status [28]. Based on differences in etoposide sensitivity (Table 3) it is not completely surprising that CHLA-10 cell line were more sensitive to morpholino-induced cytotoxicity than TC-32 cells, which have a functional P53 status [28]. While P53 status could partially explain the difference in sensitivity between EFT cell lines, recent studies suggest that variations in p16/p14 gene status may account for greater variability in drug sensitivity [28]. TC-32 cells are null for p16 and p14, while CHLA-10 cells are wild-type for both proteins, which are highly-related tumor suppressor gene products of the same cyclin-dependent kinase inhibitor 2A gene, CDKN2A. In the absence of P16 and P14, TC-32 cells may be unable to suppress G1 progression of the cell cycle, regardless of cytotoxic cues generated by therapeutic agents. These observations hint that multidrug resistance is more likely to occur in EFT cells harboring defects in p16/p14 proteins, rather than cells that have simply lost P53, which is a common artifact of drug resistant forms of EFT [28].

While oncogene status appears to dictate tumor-specific sensitivity to both conventional and gene-directed therapeutic agents, we acknowledge that cell-specific variability in antisense oligonucleotide uptake may also contribute to differences in EFT sensitivity observed. Morpholino delivery is a long-standing problem, and hence, an array of delivery strategies have been developed to improve uptake *in vitro* [31]. We utilized enhanced-delivery, Onco-morpholinos available from Onco-Tools, LLC (Philomath, OR) in combination with the PEG-based, Endo-Porter delivery reagent also available from Onco-Tools, LLC (Philomath, OR). Endo-Porter promotes the release of morpholinos from endocytic bodies that accumulate near the plasma membrane, allowing rapid and reproducible delivery of oligomers throughout the cell [32]. It is notable that CHLA-10 cells showed a greater dependence on Endo-Porter for morpholino uptake than the TC-32 cell line. For example, we achieved 27% CHLA-10 cell death with the single morpholino targeting IGFBP2-2 transcripts in the absence of Endo-Porter, and 44% cell death when Endo-Porter was employed at 10 µM (data not shown). In contrast, morpholinos targeting CYP4F22 suppressed TC-32 at similar levels (∼24%) independent of Endo-Porter (data not shown). These observations reveal significant differences among EFT cell lines, in their ability to uptake or traffic oligonucleotides across the plasma membrane, and these differences were further highlighted when administering combinatorial cocktails of morpholinos. While we lack gene expression data to explain these differences, it is possible that the passive oligomer uptake mechanisms active in TC-32 cells are disrupted by increasingly complex mixtures of morpholinos subject to both inter- and intra-molecular interactions that may disrupt import channels or trigger a compensatory efflux mechanism. Interestingly, CHLA-10 cells, which require Endo-Porter for significant morpholino uptake, were also less responsive to combinatorial cocktails at virtually all doses between 0.03–3 µM. In some cell types, Endo-Porter may be less suited to efficiently deliver complex mixtures of morpholinos, including lipid-conjugated forms (i.e. ED-morpholinos) used in this study. Despite these issues, we strongly recommend the use of Endo-Porter when developing screens for morpholino activity, *in vitro*. However, efficient delivery of complex cocktails may require further optimization of the delivery strategy, oligomer conjugation state or target-specific sequences.

It is also notable that the growth suppression observed in both EFT cell lines was highly sequence dependent for the individual morpholinos tested; insignificant levels of off-target cell death, rarely exceeding 5%, were observed when administering the scrambled control oligomer (Scr) under the same experimental conditions. A comparison of non-selective morpholino killing in a non-EFT tumor cell line (HEK293) revealed insignificant increases in cell death over scrambled control, for the majority of compounds. The net cytotoxicity (FA_24hs_) for all oligomers tested, at the most potent dose between 0.1 and 3 µM, was 13 ± 13% for HEK293 cells, compared to 17 ± 0.5% for TC-32 cells and 29 ± 14% for CHLA-10 cells (Supplementary Table 1). While we were not able to measure discrete changes in transcript or protein expression levels for the EFT-specific genes targeted in this study, it is remarkable that the average morpholino tested suppressed EFT cell growth approximately 15–43% at 24 hours, but had little to no effect on HEK293 cells, which were used in this study to compare and contrast the activity of EFT-specific morpholinos with a non-selective, topoisomerase 2 inhibitor (i.e. etoposide) in a non-EFT cell type. While HEK293 cells do not represent a normal tissue control for EFT, their similar growth rate and adhesion properties, paired with a well-characterized mechanism of immortalization (i.e. via dysregulation of pRB/p53 and apoptotic pathways) made them a reasonable, contrasting cell line for this pilot study [33]. In this regard, HEK293 cells were not used to assess off-target safety or as an activity control; the cell-specific cytotoxicity of each morpholino was controlled independently by normalizing all responses to the off-target effects observed for the scrambled control morpholino administered on the same 96-well plate. A limited amount of agent-specific toxicity was observed in HEK293 cells, but the mechanism of this off-target activity remains unknown; unexpected interactions among morpholinos and alternate pre-mRNA transcripts or other key cellular components could explain this effect. Embryonic cell types related to HEK293 cells may also have been underrepresented in our normal human transcriptome database, leading us to target some genes (e.g. CCND1 and CYP4F22) that may be overexpressed or functionally important in HEK293 cells, as well. Caution should therefore be taken when selecting RNA-Seq inputs for this approach, and control tissues should also be selected carefully based on their expected representation in the input data.

It is also notable that we identified over 10 cancer-related genes (including PHGDH, CCND1, IGFBP-2, XAGE1B/E, CYP4F22, RBM11, ORAOV1, MDK, UGT3A5, SSX5, FBL, NKX2) potentially overexpressed in EFT tumors. Each gene identified contains an exceptional 25-mer, over-represented in EFT cells at levels ∼ 10,000-fold higher than those found in any normal tissue. We expect that these target genes are highly-overexpressed in EWS tumor types, with respect to both transcript and protein level, however, it is possible that expressed forms may not be identical to those observed in normal cells due to alternative splicing. Some of the genes identified already have a well-established connection to human sarcomas, including ORAOV1 [34], SSX5 [35], NKX2 [36, 37], XAGE1 [38, 39], MDK [40] and CCND1 [41, 42]. XAGE1, in particular, is an important member of the GAGE family of cell surface protein antigens found commonly overexpressed in several human tumor types, including EWS [39]. In contrast, the human 3-phosphoglycerate dehydrogenase (PHGDH) gene, which encodes a key enzyme in the *de novo* biosynthesis of serine, was only more recently linked to EWS using an alternative, systems-based approach [43]. Our analysis also appears to link the dysregulation of RNA processing genes RBM11 [44] and FBL [45] to EWS for the first time. This harmonizes well with observations that tumors utilize alternative splicing to promote proteome diversity and oncogenic splice variant expression to promote growth, evade immune surveillance, and support tumor cell survival [46]. It is notable that synergistic CHLA-10 cell killing was observed when RBM11 was targeted in combination with CCND1. The CCND1 gene, which is directly regulated by the EWS-FLI-1 transcription factor [41], encodes two alternative transcripts of cyclin D1; the common cyclin D1a isoform and a truncated cyclin D1b variant that promotes cellular transformation and prostate cancer progression [47]. The possibility that RBM11 modulates the splicing of CCND1, and an array of oncogenic genes in EWS, is consistent with known roles for related RNA binding proteins (RBM), including RBM10, RBM15 and RBM25, which alter gene splicing to promote various forms of blood disease and cancer [48, 49]. However, because RBM11 is known to promote the alternative splicing of the pro-apoptotic, BCL-X splice variant (BCL-X_S_) [44], it remains unclear how its overexpression may contribute to complex apoptotic signaling in EFT cells.

We also linked the ceramide metabolizing enzyme, CYP4F22, to EFT cell types for the first time. CYP4F22, an orphan P450 gene associated with hyperkeratotic skin diseases [50, 51], is now recognized as the fatty acid ω-hydroxylase gene required for acylceramide production in skin [52]. The Cancer Genome Atlas links CYP4F22 overexpression to hepatocellular carcinomas (found in ∼60% of samples tested) and tumors of the bladder, breast and ovaries, however its role in cancer progression remains unclear [53]. We hypothesize that CYP4F22 overexpression may function to regulate the accumulation of pro-apoptotic sphingolipids (e.g. ceramide) in cancer cells, which similar to RBM11, promote alternative splicing of the BCL-X_S_ splice variant [54, 55]. Exaggerated CYP4F22-mediated metabolism could potentially reduce cellular ceramide levels below what is required for pro-apoptotic signaling and promote tumorigenesis. In this regard, CYP4F22 overexpression may function similar to the acid ceramidase gene (AC), overexpressed in multiple tumor types [56], and increasingly recognized as an important therapeutic target for pediatric brain tumors [57], acute myeloid leukemia [58], and melanoma [59, 60]. Ceramide modulators were reported to enhance the cytotoxicity of Fenretinide, a p38 MAP kinase activator drug, in EWS cells [61], and CYP4F22 inhibition strategies may be worth consideration in combination therapies with chemotherapeutics, like cisplatin, which has shown enhanced cytotoxicity when co-administered with an acid ceramidase inhibitor [62].

Our study also associates the dysregulation of the phase II drug-metabolizing gene, UDP glycosyltransferase 3A2 (UGT3A2) to EWS for the first time [63]. Dysregulation of UGT family pathways was also previously reported in castration-resistant forms of prostate cancer [64]. UDP glycosyltransferases (UGTs) detoxify lipophilic small molecules, and it is possible that their overexpression may also modulate the fate of pro-apoptotic signaling molecules, like ceramide.

Another notable observation derived from our EWS-specific transcriptome profiling is the potential overexpression of the insulin growth factor binding protein, type 2 isoform (IGFBP-2). Insulin-like growth factor (IGF) signaling promotes tumorigenesis and the EWS/FLI-1 transcription factor induces the IGF receptor (IGF1R) gene, while concomitantly suppressing expression of the insulin-like growth factor binding protein 3 (IGFBP-3) gene; the latter binds insulin to silence IGF1R signaling [65]. The downregulation of IGFBP-3 is a hallmark of the EWS phenotype [66], but the role of IGFBP-2 overexpression appears unresolved. Our results highlight a regulatory differences in IGBFP-2 and IGFBP-3 gene activation, which may coordinately regulate IGF1R signaling. We detected an over-represented k-mer in four splice variants of the IGFBP-2 gene (see Supplementary Figure 1), however our approach did not discriminate if the same k-mer was found in all splice variations of the gene. Further analysis of IGFBP-2’s functional role and variant-specific expression profile in EWS is needed, but based on our analysis it may represent a novel therapeutic target for EWS similar to related EWS-FLI1 related genes, including the Aurora kinase (AURKA) and the cholecystokinin (CCK) receptor [67].

In summary, k-mer-based profiling identified 12 genes potentially overexpressed in the EWS-specific transcriptome. Antisense-oligonucleotide mediated EFT cytotoxicity evaluations suggest single agent or combinatorial drug regiments targeting these genes may hold promise for EFT treatment strategies, and contribute to the expanding number of gene-targeted therapies for various sarcomas [68]. Here, we have described a simple method to rationally identify and parse a precision set of tumor specific gene targets, and rapidly assess their cytotoxicity using a high-throughput morpholino-based assay. Mechanistic insights are still required to explain the cytotoxic effects we observed, but the identification of RBM11, CYP4F22 and IGFBP-2 as new potential drug targets for EFT would seem to confirm the utility of this open source, computational methodology for cancer drug discovery. Our results also clarify the rigor required to develop precision-based, combinatorial therapeutic cocktails for cancer, which are promising, but potentially ineffective as they can induce both synergistic and antagonistic drug interactions [69]. Despite limitations to this approach, we have identified several single agents that are highly cytotoxic to EFT cells, and morpholino cocktails targeting PHGDH transcripts, in combination with XAGE1 or CYP4F22, may be highly synergistic across multiple EFT tumor types.

In conclusion, the k-mer-based computational approach developed here is an effective transcriptomic-based methodology for identifying new targets for the treatment of human cancers. Limitations of this method include the availability of open source RNA-Seq data for a particular malignancy, and the quality and completeness of comparative transcriptomic data, which should account for human diversity and variations in gene expression across multiple cellular sub-types and stem cells. This approach represents a feasible approach for precision-based cancer therapy that compares a patient’s personalized cancer transcriptome with open source healthy controls. Synergistic combinations of morpholinos directed at cancer-specific gene targets hold significant potential to rapidly identify new, highly effective anticancer therapies (HEAT).

## MATERIALS AND METHODS

### Reagents

RPMI and Iscove’s DMEM media and additives were obtained from Sigma (St. Louis, MO). Fetal Bovine Serum was obtained from Atlanta Biologics (Flowery Branch, GA). Presto Blue Cell Viability reagent was obtained from ThermoFisher Scientific (Waltham, MA). Etoposide was obtained from Cayman Chemical Company (Ann Arbor, MI). Onco-morpholinos and Endo-Porter delivery reagent was obtained from Onco-Tools, LLC (Philomath, OR).

### K-mer-based identification of gene targets

RNA-Seq data from three Ewing’s Sarcoma (EWS) cell lines (TC-32, A673, and TTC-466) provided core sequence information for the k-mer selection strategy (see details below). A k-mer-based subtraction analysis method for identifying exceptional genes among three, prototype EWS cell lines and 26 normal healthy human tissues identified 1.09 × 10^8^ potential gene targets containing tumor-specific k-mers (Table 1). Using the down selection strategy highlighted in Table 2, we identified 6 lead gene targets for antisense drug development, each with an anticipated independent mechanisms of action against EWS tumors. Transcripts for these 6 genes were found to be highly-overrepresented in the 3 EWS cell lines, compared to the 26 healthy tissues sampled (Table 2).

### K-mer counting strategy

25-mers were cataloged with Jellyfish software (version 2.2.3) using data from the Updated Human Protein Atlas RNA-Seq dataset available in the ArrayExpress database (http://www.ebi.ac.uk/arrayexpress) under accession number: E-MTAB-1733 [16–18]. The k-mer abundance for this dataset, representing 26 different tissues from 95 individuals, with between 6 and 26 samples per tissue, was compared to a cancer-specific RNA-Seq dataset comprised of three Ewing’s Sarcoma RNA-Seq samples (Cell lines/SRA archive accessions: A673/SRR2541170, TC-32/SRR2541171, TTC-466/SRR2541172) available in the ArrayExpress database under accession number: E-GEOD-73610 [16, 19]. Due to Jellyfish constraints, k-mers present less than 10 times in a sample were not counted for that sample. K-mers not counted amongst any sample were not considered for analysis (counted as 0 throughout). For a conservative measure of abundance for targeting, k-mers represented below the 10-count-threshold were input as 0 for Ewing’s Sarcoma samples, and 10 for normal-tissue samples.

Each per-sample k-mer count provided by Jellyfish was normalized to sample size (i.e. to count-per-billion-bp of sequencing effort), producing a per-sample “k-mer abundance.” K-mer filtering efforts considered the ratio of k-mer abundance in Ewing’s sample to those in normal samples on a per-tissue basis, taking the smallest abundance in Ewing’s samples to the largest abundance amongst normal tissue samples. For example, if k-mer A had abundances in Ewing’s samples of 28341, 349248, and 54922, and abundances in normal cerebral cortex samples of 34115, 1551, 16368, 54135, 167743, and 2376, the abundance ratio for this k-mer between these tissues was 28341/167743 = 0.16. K-mers with abundance ratio > 500 in a given tissue were considered “over-abundant” for that tissue.

Simultaneously, all 25-mers present in the known human transcriptome (Ensembl GRCh37; hg19 [20]) were cataloged, including their locations within known protein coding genes and non-coding RNA transcripts. For each human transcript, we identified the contained k-mer that was over-abundant (ratio > 500) for the maximum number of tissues, associating that “maximum over-abundance tissue count” and “maximum over-abundance tissue k-mer” with the transcript. The abundance ratios for top 400 transcripts in this regard were plotted in a heat map visualizing, for each tissue/transcript combination, the abundance ratio for the selected k-mer across 26 tissues (Supplementary Figure 1). For all of these selected k-mers, we reconsidered the original counts in the Jellyfish output, and found them all to be 0 in normal samples (and thus imputed to 10).

### Oligomers

6 EFT-specific genes were selected for antisense targeting using the k-mer-based transcriptomics approach, and morpholinos targeting multiple transcript forms are listed in Table 2 along with their anticipated biological significance. The oligomers were synthesized as Onco-Morpholinos by Onco-Tools, LLC (Philomath, OR) (https://www.onco-tools.com/about-our-products). Morpholinos produced by Onco-Tools are considered “reagent grade”; each oligomer generated is accompanied by a specification sheet which includes, a reference number, order number, date of order, sequence, molecular weight, molar absorptivity at 265 nm in 0.1 HCl, weight in mg produced, OD units (265 nm, pH1), skeletal molecular structure of the morpholino, and MALDI-TOF mass spectral analysis of the product to demonstrate removal of waste products by selective precipitation (synthesis resin, ammonia, cleaved base-protective groups, and minor amounts of short truncation fragments). Enhanced delivery Onco-morpholinos contain a proprietary fatty acid conjugated to the 3′ terminal amine group that enhances oligomer cellular uptake when used in combination with the delivery agent Endo-Porter (10% PEG 1500/H20 formulation).

### Cell culture

TC-32, TC-71, CHLA-9 and CHLA-10 cells were obtained from the Children’s Oncology Group Cell Culture and Xenograft Repository at Texas Tech University (http://www.cogcell.org/index.php). TC-32 cells were grown following COG recommendations in a base medium of RPMI-1640 plus 10% fetal bovine serum and 2 mM L-Glutamine. TC-71, CHLA-9 and CHLA-10 cells were grown as advised in a base medium of Iscove’s Modified Dulbecco’s Medium supplemented with 20% fetal bovine serum, 4 mM L-Glutamine, 1X ITS (5 ug/mL insulin, 5 ug/mL transferrin, and 5 ng/mL selenous acid). All cell lines were pre-validated with original patient samples using short tandem repeat (STR) analysis.

### Ewing’s sarcoma cell viability assay method

The tumor cell proliferation assay protocol used here was adapted from several standard MTT assay protocols [21–23] but employs the PrestoBlue^®^ Cell Viability Reagent (#A13261; Invitrogen, Carlsbad, CA), which was utilized according to the manufacturer’s instructions. On day one, 24 hours prior to oligonucleotide treatment, TC-32 and CHLA-10 Ewing Sarcoma cells were seeded into 96-well plates (µClear^®^ White Flat Bottom; #655098, Greiner Bio-One; Monroe, NC) and incubated at 37C (5% CO2) overnight. Cells were first counted in a hemocytometer and diluted to 750,000 cells/mL in media. Next, 7,500 cells were added to each well in a final volume of 100 uL. On day two, the media was replaced, and cells were treated with fresh media containing an experimental Onco-Morpholino, a morpholino cocktail (0.03–3 µM total oligomer) or the standard scrambled control Onco-morpholino (Onco-Tools, LLC, Philomath, OR). Cellular uptake of Onco-morpholinos was induced using Endo-Porter (Onco-Tools, LLC), which was used according to the manufacturer, at a final concentration of 10 µM, after toxicity screening in individual cell lines (5–20 µM Endoporter; 24 hrs). TC-32 cells require RPMI media (10% FBS; 1X Glutamine) and CHLA-10 cells require Isocove’s DMEM media (20% FBS, 1X ITS, 4 mM Glutamine) for general culturing. However, when screening cell viability in the presence of Endo-Porter, serum was reduced in standard media to 2% for both cell types. The same conditions were used when determining benchmark toxicity doses for etoposide. On day 3, at ∼24 hours post-morpholino treatment, media was removed from cells, and 100 uL of PrestoBlue^®^ Cell Viability Reagent (Invitrogen; A13261) was added to each well, and the plate was incubated for an additional 15 minutes at 37C (5% CO2). At 30 minutes post-addition, fluorescence was measured in each well using a Synergy 2 Multimode plate reader (Biotek); absorbance was monitored between 535–560 nm, with a reference wavelength between 590–615 nm (570 nm absorbance maxima, with 600 nm reference, recommended). Each experimental condition was assessed with at least 5 biological replicates (*N* = 5).

### Measure of fraction dead cells

The greater the resorufin signal, the more living cells in the culture. The Fraction Dead Cells, measured between 0.00 and 1.00, is calculated using the equation:

$$\text{Fraction Dead Cells}=\left({\text{Resorufin}}_{\text{Control}}{-\text{Resorufin}}_{\text{Treated}}\right){/\text{Resorufin}}_{\text{Control}}.$$

### Compusyn synergy analysis

CompuSyn utilizes the Chou-Talalay method for studying multidrug combinations; this approach is based on the median-effect equation, derived from the mass-action law principle, which is a unified theory linking single agent effects to multi-agent effects, providing the theoretical basis for deriving quantitative Combination Index (CI)-isobologram equations needed to compare the combined effects of two or more drugs [24]. The log concentration versus fraction dead cells relationship was evaluated using Excel fitting data to a linear regression line and inspection of the slope, intercept, and r^2^ values. Confirmation of median effect concentration (Dm) and calculation of the combination index (CI) was done with CompuSyn software based on a published review [24]. CI values of 1 indicate summation of effect (or additive effect), CI > 1 indicates antagonism in the combination, and CI < 1 indicates synergism in the combination.

### Statistical analysis

At least six replicate culture wells were evaluated for each treatment condition. The mean fraction dead cells from each the log_10_ morpholino treatment concentrations (0.01, 0.03, 0.1, 0.3, 1, and 3 µM) were modelled by linear regression using Excel software (Microsoft, Redmond, WA) and the correlation coefficient and slope were calculated. The slope different from zero was considered significant at *p* ≤ 0.05 to confirm a dose dependent response. Singe agent efficacy (FA_24 hrs_) was evaluated by ANOVA analysis paired to a student *T*-test.

## SUPPLEMENTARY MATERIALS FIGURE AND TABLE



## Abbreviations

EFT: Ewing’s Family Tumor
EWS: Ewing’s Sarcoma
CI: combination index
Dm: median effect concentration
RNA-Seq: RNA sequencing
HTS: high-throughput sequencing
T:N ratio: tumor-to-normal ratio
FA_24hs_: fraction cells affected at 24 hours
PNET: primitive neuroectodermal tumors
XAGE1: X antigen family member 1
CCND1: Cyclin D1
RBM11: RNA binding motif protein 11
CYP4F22: Cytochrome P450 family 4 subfamily F member 22
PHGDH: Phosphoglycerate dehydrogenase
IGFBP-2: Insulin like growth factor binding protein 2
