# Supplementary material for: A k-mer based transcriptomics approach for antisense drug discovery targeting the Ewing’s family of tumors
Source: Oncotarget. 2018 Jul 17;9(55):30568–86. doi: 10.18632/oncotarget.25736 (PMC6078127; doi:10.18632/oncotarget.25736)
Supplement: Supplementary file 1 [file oncotarget-09-30568-s001.pdf]

A k-mer based transcriptomics approach for antisense drug discovery targeting the Ewing's family of tumors

SUPPLEMENTARY MATERIALS

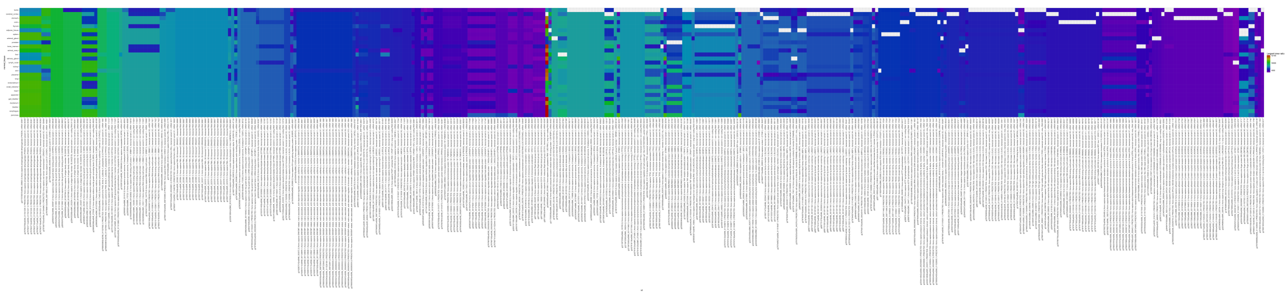

Supplementary Figure 1: High-resolution heat map analysis of the EWS-specific transcriptome. The k-mer-based transcriptomics method developed here identified 1.09 × 10^8 mRNA transcript fragments (25 bases) over-represented in 3 EWS cell lines (A-673, TC-32 & TTC-446) at levels 500-fold above those found in any of 26 normal human tissues. Over-abundant k-mers were mapped to individual protein-coding and non-functional RNA transcripts in the human genome (GRCh37; hg19) and a heat map was generated for the top 400 transcripts overexpressed at tumor-to-normal (T:N) levels exceeding 1000:1 (shown in blue) across the most tissues. Exceptional transcripts with T:N ratios exceeding 10,000:1 (shown in green) were evaluated for their known roles in tumorigenesis and their links to EFT tumors, and 6 of 12 primary targets identified were down-selected for further evaluation as potential therapeutic targets for EFT using an antisense-based, reverse-genetics approach. Our preliminary analysis of the EWS transcriptome highlights the potential of meta-transcriptomics approaches to rapidly identify novel gene targets for human therapy using only open source RNA-seq data. Multiple EWS-specific genes listed in our heat map (with T:N ratios > 1,000) may serve important functional roles in ETF tumorigenesis, however further analysis of their expression in the disease state are required to assess their potential as therapeutic targets for the treatment of EWS and related EFT malignancies.

Supplementalry Table 1: Maximum single agent activity† at 24 Hours

	HEK293 cells	TC-32 cells	CHLA-10 cells
**Antisense agent**	**Fraction dead cells at 24 hours $(FA_{24\ hs})_{corrected}$†**		
**XAGE1E**	0.13	0.18	0.53
**CCND1**	0.05	0.10	0.39
**RBM11**	0.02	0.12	0.46
**CYP4F22-1**	0.02	0.12	0.10
**CYP4F22-2**	0.38	0.17	0.21
**CYP4F22-3**	0.12	0.24	0.03
**PHGDH-1**	0.21	0.21	0.23
**PHGDH-2**	0.35	0.17	0.33
**IGFBP2-1**	0.04	0.15	0.44
**IGFBP2-2**	0.25	0.25	0.27
**IGFBP2-3**	0.04	0.12	0.29
**IGFBP2-4**	0.12	0.17	0.21
**Average ± SD**	$FA_{24\ hs} = 0.13 \pm 0.13$	$FA_{24\ hs} = 0.17 \pm 0.05$	$FA_{24\ hs} = 0.29 \pm 0.14^{*}$

†Activity or Fraction Dead Cells at 24 hours $(FA_{24\ hrs})_{corrected}$ was calculated for single agents using the fraction of cells lost among 5 replicate samples at the most potent morpholino dose between 0.1 and 3 μM, corrected to the average fraction of cells lost when treated with the same concentration of the scramble control morpholino; All replicates were measured on the same 96-well tray and all wells were seeded with an identical number of starting cells, 24 hours prior to treatment.
$^{*}p < 0.05$, based on paired student *t*-test comparing mean corrected activities of individual morpholinos in the HEK293 control cell line.
